# Percolation Centrality: Quantifying Graph-Theoretic Impact of Nodes during Percolation in Networks

**DOI:** 10.1371/journal.pone.0053095

**Published:** 2013-01-22

**Authors:** Mahendra Piraveenan, Mikhail Prokopenko, Liaquat Hossain

**Affiliations:** 1 Centre for Complex Systems Research, Faculty of Engineering and IT, The University of Sydney, New South Wales, Australia; 2 CSIRO Information and Communications Technology Centre, Epping, New South Wales, Australia; 3 School of Physics, The University of Sydney, New South Wales, Australia; Umeå University, Sweden

## Abstract

A number of centrality measures are available to determine the relative importance of a node in a complex network, and betweenness is prominent among them. However, the existing centrality measures are not adequate in network percolation scenarios (such as during infection transmission in a social network of individuals, spreading of computer viruses on computer networks, or transmission of disease over a network of towns) because they do not account for the changing percolation states of individual nodes. We propose a new measure, *percolation centrality*, that quantifies relative impact of nodes based on their topological connectivity, as well as their percolation states. The measure can be extended to include random walk based definitions, and its computational complexity is shown to be of the same order as that of betweenness centrality. We demonstrate the usage of percolation centrality by applying it to a canonical network as well as simulated and real world scale-free and random networks.

## Introduction

Networks are ubiquitous in today's world. Communication networks such as world wide web, telephone networks and mobile phone networks are changing the way we live and we interact with other people. Social networks built on top of these, such as Facebook and Twitter, are redefining ways of keeping in touch. Vast airline and rail networks have given us access to the remotest parts of the world and reduced travel times by orders of magnitude. Our survival depends on the functioning of a number of biological and ecological networks. The energy needed for our domestic and industrial use is supplied by electric power networks. Indeed, the interest and awareness about networks are not only a trend in scientific research but also a social and cultural phenomenon of this age [1]–[5].

Percolation of a ‘contagion’ occurs in complex networks in a number of scenarios. For example, viral or bacterial infection can spread over social networks of people, known as contact networks. The spread of disease can also be considered at a higher level of abstraction, by contemplating a network of towns or population centres, connected by road, rail or air links. Computer viruses can spread over computer networks. Rumours or news about business offers and deals can also spread via social networks of people. In all of these scenarios, a ‘contagion’ spreads over the links of a complex network, altering the ‘states’ of the nodes as it spreads, either recoverably or otherwise. For example, in an epidemiological scenario, individuals go from ‘susceptible’ to ‘infected’ state as the infection spreads. The states the individual nodes can take in the above examples could be binary (such as received/not received a piece of news), discrete (susceptible/infected/recovered), or even continuous (such as the proportion of infected people in a town), as the contagion spreads. The common feature in all these scenarios is that the spread of contagion results in the change of node states in networks.

Indeed, in the epidemiological domain, a few studies have successfully modelled epidemic spread as a specific example of percolation in networks [6]–[11]. The percolation theory is attractive because it provides connections to several well-known results from statistical physics, in terms of percolation thresholds, phase transitions, long-range connectivity, and critical phenomena in general. For instance, Newman and Watts [6] suggested using a site percolation model for disease spreading in which some fraction of the population is considered susceptible to the disease, and an initial outbreak can spread only as far as the limits of the connected cluster of susceptible individuals in which it first strikes. An epidemic can occur if the system is at or above its *percolation (epidemic) threshold* where the size of the *largest (giant) cluster* becomes comparable with the size of the entire population. Similarly, Moore and Newman [12] used a general model with two simple epidemiological parameters: (i) *susceptibility*, the probability that an individual exposed to a disease will contract it, and (ii) *transmissibility*, the probability that contact between an infected individual and a healthy but susceptible one will result in the latter contracting the disease. They pointed out that if the distribution of occupied sites or bonds is random, then the problem of when an epidemic takes place becomes equivalent to a standard percolation problem on the graph: what fraction of sites or bonds must be occupied before a “giant component” of connected sites forms whose size scales extensively with the total number of sites [12]. It has been noted [13] that the percolation of disease through a network depends on both the level of contagion and the structure of the contact network. Similarly, in other contexts such as virus spreading in a computer network or information spreading in a social network, it could be deduced that percolation is determined by both network topology and amount of the contagion spreading.

In any context, if we need to stop the contagion from spreading further, we need to supply nodes with certain resources. For example, during a disease outbreak affecting a network of towns, medical staff, medicine and other resources need to be rushed to each town to stop the infection from spreading to other towns as well as to treat people in that town. Generally, there are limited resources (vaccines, drugs, medical staff, transport, etc.) to respond in time. Therefore, choices for early intervention in the affected network need to be precise. However, ‘nodes’ that are individually at the highest risk are not necessarily those which will contribute most to the contagion transmission. Hence, there is a need to identify nodes that are ‘central’ in terms of their impact on the spread. Moreover, we need to interpret the node's impact both in terms of their topological connectivity and their current infected (percolated) state. Intuitively, an infected node makes a higher impact than a non-infected one even if their topological connectivity is identical. Furthermore, different levels of risks, susceptibility, etc. can bring about non-binary node states making the assessment of the impact even less trivial.

We may formulate the scenarios described above into a general problem: In a given complex network, to what extent the individual percolated (or partially percolated) nodes impact on the percolation process at any given time? A measure quantifying this extent needs to not only account for topological connectivity but consider the node's percolation state (including partial percolation). The existing centrality measures are not adequate for this purpose because they do not account for changing percolation states of individual nodes, and are static. Therefore a suitable centrality measure which also takes into account the percolation states of nodes is needed, which should be general enough to be applicable in all the contexts described above.

In this paper, we introduce a new centrality measure, *percolation centrality*, capturing relative impact of nodes during (possibly partial) percolation. The proposed measure subsumes betweenness centrality by explicitly accounting for percolated nodes on relevant shortest paths. When all nodes are in the percolated state this measure is shown to be equivalent to betweenness centrality. We also briefly analyze the computational complexity of percolation centrality showing that it is of the same order as that of betweenness centrality. Furthermore, we succinctly discuss possible extensions to random walk based definitions of this measure. We employ *generic scale-free networks* to analyze percolation centrality, since it has been shown that a great number of real world networks, including contact networks, social networks of people in general, transport networks, and large scale computer networks (including Internet), tend to be scale-free [1], [2], [14]–[21]. We also analyze random networks for comparison. We employ a simulation approach to illustrate how the measure of percolation centrality could be used as a tool in intervention strategies, by comparing it to betweenness centrality and shortest distance from percolated nodes. Finally we present our observations and conclusions.

## Analysis

### Review of centrality and network evolution measures

A host of centrality measures have been proposed to analyze complex networks, especially in the domain of social network analysis. The simplest of these perhaps is the degree centrality, sometimes just called degree, of a node. A node's degree is simply the number of links it has with other nodes in the network, and therefore gives some indication about how important that node is to the network.

A family of betweenness measures have been proposed [22]–[28] to measure a node's importance as a conduit of information flow in a network. The first and perhaps most well known measure of these is the classical betweenness centrality measure proposed by [22]. Betweenness Centrality measures the fraction of shortest paths that pass through a given node, averaged over all pairs of node in a network. It is formally defined, for a directed graph, as
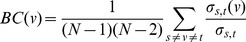
(1)where 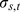 is the number of shortest paths between source node 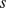 and target node 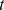, while 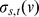 is the number of shortest paths between source node 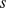 and target node 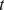 that pass through node 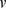.

Closeness Centrality [23], [29] is a measure of how close a network is, on average, to the rest of the nodes in terms of shortest paths. It essentially measures the average geodesic distance between a given node and all other nodes in the network. It is defined as
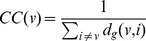
(2)where 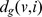 is the shortest path (geodesic) distance between nodes 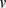 and 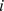. Note that the average is ‘inverted’ so that the node which is ‘closest’ to all other nodes will have the highest measure of closeness centrality.

The Eigen vector centrality measure [30] is based on the assumption that a node's centrality is influenced by the centrality scores of its neighbours - that the centrality score of a node is proportional to the sum of the centrality scores of the neighbours. As such, it is defined iteratively. If the centrality scores of nodes are given by the matrix X and the adjacency matrix of the network is A, then we can define x iteratively as
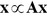
(3)i.e
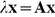
(4)


The centrality scores are obtained by solving this matrix equation. It can be shown that, while there can be many values for 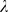, only the largest value will result in positive scores for all nodes [31].

The classical betweenness centrality measure assumes that information flow is through the shortest paths in a network. This is, in many instances, not a realistic assumption [24], [26], [27]. For example, in a transport network, the traffic will likely go through alternative paths if the shortest path is traffic-jammed. Rumours or infections in social networks are likely to follow random paths. A number of centrality measures based on betweenness address this. The flow centrality measure [22] measures the proportion of the ‘flow’ that goes through a given node, when maximum flow is ‘pumped through’ a pair of nodes. A random walk based betweenness measure proposed by Newman [26] considers a network to be like an electric circuit with unit resistance at any link, and measures the ‘current’ that goes through a node when unit current passes through a pair of nodes (In fact, the random walk based betweenness is not formally defined in this way. However, as mentioned in [26], it is the easiest way to intuitively understand this betweenness measure.).

There are a number of other centrality measures based on random walks as well, such as those described in [32], [33]. There is also the information centrality measure [27] based on closeness centrality, which measures the harmonic mean length of paths ending at a vertex 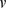. The power centrality [26], [33] of a node 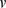 is the number of times a random walk is expected to pass through the node 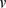, averaged over all possible starting points of the random walk. The random-walk centrality introduced by [32] measures the average speed with which, a randomly walking message from a node reaches the target node 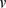, averaged over all source nodes.

A number of weighted betweenness measures, such as [28], where weights are given to links, have also been proposed recently. Klemm et al. [34] introduced another measure, *dynamical influence*, to assess the role of an individual in collective dynamics within a system of interacting elements. Dynamical influence quantifies the extent to which an initial condition of a specific node affects its final state, given the system dynamics. The new measure was applied in an epidemiological scenario, and was shown to be a good predictor of spreading efficiency in a social network (spreading efficiency measures the expected fraction of nodes reached by an epidemic outbreak initiated with a specific infected node). Dynamical influence, however, estimates the influence of a node on a potential spread before the contagion begins — it is a measure of inherent dynamics of which the system is potentially capable. It is not, however, a time-dependent measure of actual dynamics which is affected by current state of a node.

Another method to efficiently approximate the number of infections resulting from a given initially infected node in a network of susceptible individuals is described by Bauer and Lizier [35]. While this method directly considers the spreading process and provides an estimation of actual numbers of infections, it is also aimed at estimating the impact of the initial infected node on the infection spreading, rather than a time-dependent impact of any other node on the percolation in the network.

There are a number of measures that do characterise individual nodes at every step of network evolution. For example, a prominent approach to modelling cascading failures is based on a study of Goh et al. [36] and Crucitti et al. [37]. The model proposed by the latter group, Crucitti-Latora-Marchiori (CLM) model, has also been extended [38]–[40] to studies of cascading failures in power grids. In these studies, a power grid is represented as a weighted graph, and each node is characterised by a load (e.g., electrical load) which varies over time and has a fixed limited capacity. The load is defined as the number of most efficient paths (e.g., from generators to distribution substations) that pass through that node. Original CLM model considered paths between all node pairs, and the load was equivalent to weighted betweenness centrality. Consequently, the more shortest paths pass through a node, the higher is its load. A cascading failure scenario is triggered by a (random) failure of a single node, affecting its neighbours as well as relevant shortest paths, and therefore redistributing the load. When capacity of any affected node is exceeded by its new load, the overloaded node also fails, and the cascade of failures may continue. In short, the node's load is a time-dependent property. It is important to point out that overloaded nodes are not removed from the network (apart from the very first point of failure) — instead, the efficiencies of links connecting to each overloaded node are reduced in proportion to the overload. This in turn changes weighted shortest paths. That is, the changes in loads (i.e., their weighted betweenness centralities) are brought about by recalculation of shortest paths. In other words, the changes are topologically-driven, rather than being reflections of new states of nodes which remain connected in the same way.

In addition, a particular probabilistic routing scheme may be assumed instead of shortest path routing or Newman's random walk routing. This notion is generalized in another study that discussed Routing Betweenness Centrality [41]. In these variants betweenness centrality calculations assume that traffic flows over shortest paths, but use a different routing mechanism. These variants are time-independent: e.g., routing betweenness centrality of a node does not change over time, and hence does not characterise an impact that a percolated/infected node has on the network.

Further classification of measures was carried out by Borgatti and Everett [42]. In particular, their review distinguished between radial and medial measures. For example, measures that assess walks that emanate from or terminate with a given node are called radial, while the measures which are based on the number of walks that pass through a given node are called medial measures.

### Percolation centrality

As described in the previous section, a slew of centrality measures exist to determine the ‘importance’ of a single node in a complex network. However, these measures quantify the importance of a node in purely topological terms, and the value of the node does not depend on the ‘state’ of the node in any way. It remains constant regardless of network dynamics. This is true even for the weighted betweenness measures [28]. However, a node may very well be centrally located in terms of betweenness centrality or another centrality measure, but may not be ‘centrally’ located in the context of a network in which there is percolation. With this in mind, we propose percolation centrality (PC), which specifically measures the importance of nodes in terms of aiding the percolation through the network.

Let us denote the percolation state of node 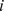 at time 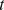 by 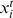. When the temporal context is clear we shall simply use 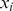. Specifically, 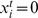 indicates a non-percolated state at time 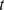, 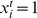 indicates a fully percolated state at time 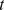, while a partially percolated state corresponds to 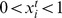 (e.g., in a network of townships, this would be the percentage of people infected in that town). The higher the value 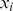, the higher is the degree of percolation of node 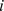. In this study, we do not discuss how precisely the node states are assessed or assigned, since that is context-dependent, and assume that a mechanism for quantifying the levels of partial percolation exists. Rather we focus on determining, at any time, how important is the node to the overall process of percolation.

We define percolation centrality for a given node, at a given time, as the proportion of ‘percolated paths’ that go through that node. A ‘percolated path’ is a shortest path between a pair of nodes, where the source node is percolated (e.g., infected). The target node can be percolated or non-percolated, or in a partially percolated state. As an extension, we will later consider the case where the target node has to be specifically non-percolated (e.g., non-infected). Formally, percolation centrality of node 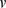 at time 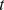 is:
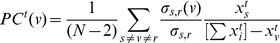
(5)where 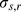 and 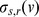 are defined as usual.

Let us examine the fraction
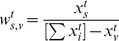
(6)in more detail, as it captures the relative contribution (weight) of each percolated path originated in the source node 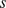 to the percolation centrality 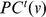.

The percolation state associated with a source determines how much importance is given to the potential percolation paths that originate from it. The sum in the denominator is the total extent of percolation in the network, ranging from zero when there are no percolated nodes, to 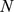 when all nodes are fully percolated. The state of node 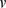 is subtracted from the total to ensure the proper normalisation (Since each percolated node can have 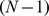 targets, we need to normalize the weights of these nodes so that they will add up to 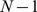, and the average path has a weight of unity.):
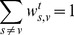
(7)The node 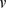 itself can be either counted or not counted as a source/target node in the definition of betweenness centrality [26] and consequently, in the definition of percolation centrality. We have adopted the convention of not counting 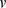 as a source or target node.

Obviously, if 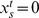 the contribution 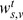 is zero, and the source 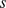 is not contributing to PC. In particular, when there are no percolated nodes, the percolation centrality is trivially zero. The first percolated node, however, will affect the PC of multiple nodes, resulting in the average PC of these nodes being significantly higher than the average betweenness of these nodes. In fact, it is possible to show that if only one node 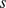 is infected (or partially percolated to the extent 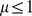) then for any other node 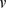


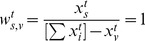
(8)and hence
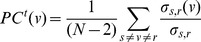
(9)This in turn means that if we iterate over all possible *single* nodes 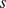 infected to the same level 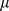 (that is, consider all possible contagion sources one by one), and then average over all these scenarios, we obtain the average percolation centrality of the node 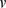 in the face of all 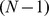 possible contagion origins:
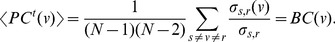
(10)That is, percolation centrality averaged over all possible single contagion sources reduces to betweenness centrality.

Finally, if all nodes are fully percolated (or partially percolated to the same extent 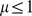) and 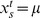 for all possible sources as well as the node 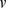 itself, the contribution 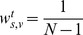 is constant at that time, resulting in
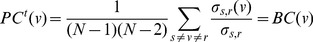
(11)In this case all shortest paths become percolated paths, since all nodes are potential ‘sources’ of percolation. It is evident then that during the process of percolation, the PC is significantly different from betweenness at 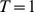 for nodes near the infection, and converges to 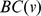 over time. It may be conjectured that on average (across the nodes) such a convergence will undergo a sharp transition, resembling a phase transition expected during network percolation. That is, when the size of the giant percolated cluster becomes comparable with the size of the entire population, the average PC becomes comparable with average BC.

We would like to note that the matrix of weights 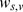 is easily obtainable, at any time, as
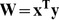
(12)where 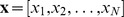, and elements of the 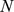-dimensional vector 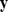 are defined as
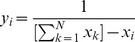
(13)In other words, the rows of 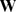 correspond to the source nodes, and the columns correspond to the nodes for which the percolation centrality is being calculated.

We attached weights to the percolation paths depending on the percolation levels assigned to the *source* nodes, based on the premise that the higher the percolation level of a source node is, the more important are the paths that originate from that node. Nodes which lie on shortest paths originating from highly-percolated nodes are therefore potentially more important to the percolation. One may then ask whether the percolation level of target nodes need to be accounted for. Does the percolation level of a target node also determine the importance of shortest paths leading to it? This depends on the context of the application. For example, in the case of spread of infection, over social networks of people or networks of towns, one may argue that if the source node and target node have equal levels of percolation (infection), then the paths connecting them are insignificant. Indeed, in some contexts, a path is meaningful as a potential path of percolation (infection) spread only if the source node is at a higher level compared to the target node.

With this in mind, we may extend our definition of percolation centrality to include target node weights. In this case, we have to calculate path weights as a difference of source node weights and target node weights, and we set the path weight to zero if this difference is negative. Therefore, there will be 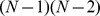 pairs of nodes, and the total weights of paths between all nodes have to sum up to 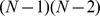 so that the average path weight will be unity. Thus our definition for percolation centrality, at time 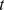, taking into account both source and target node weights, becomes
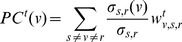
(14)where the weights are given by
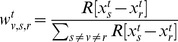
(15)using the Ramp function 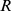, defined as 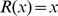 for positive 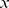 and 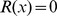 for negative 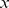. The scaling factor 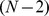 has disappeared (compared to equation (5)) because it has been absorbed into the weights.

Further analysis of PC in this study is based on the weights of source node states alone. Source-target weight based percolation centrality will be discussed in a follow-up paper.

Let us note here that, while a number of weighted betweenness centrality measures already exist, such as [28], the measure we propose here is subtly different. We do not compute importance of paths based on weights of links, but based on *states* of nodes. As such, our measure is dynamic, whereas the existing weighted centrality measures are static. Even though links in our context could be interpreted as having weights, these weights are inherited from node states of source and target, do not depend on the intermediate nodes, and will change with time.

Revisiting some of the measures briefly reviewed in previous section, we note the following differences. Dynamical influence [34] assesses the influence of a node on a potential spread before the percolation begins. Hence it is not a time-dependent measure of actual dynamics, unlike the proposed percolation centrality which is affected by current states of a node.

The family of CLM models introduced time-dependence. However, the changes in the weighted betweenness centralities of nodes (their loads) occur because shortest paths are recalculated at each step due to new link efficiencies (edge weights). As pointed out in previous section, these changes do not account for dynamics of new nodes' states: betweenness centrality changes because shortest paths are different. On the contrary, percolation centrality changes because the nodes' states are updated while the shortest paths remain the same.

It is interesting to point out at this stage that percolation centrality is a hybrid measure in terms of Borgatti and Everett classification [42]: it is a medial measure because it utilises shortest paths, following betweenness centrality, and it is also a radial measure because it assesses the state of the sources (and targets).

Finally, one may argue that percolation centrality is a routing betweenness centrality variant in which time-dependence is added via percolating states of (infected) nodes, rather than updating efficiencies of links.

In addition, the proposed measure accounts for partial percolation (a node state may take any value between, say 0 and 1), and can be applied with an immunization focus: which nodes need to be immunized first, rather than with the focus on the spreading efficiency: what is the average outbreak size if the contagion originates at a specific node.

Let us also define ‘Hop distance’ while we discuss percolation centrality. Hop distance of a node 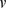 regarding node state 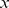, 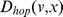, is simply the shortest distance from a given node 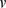 to any node with a particular state 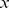. For example, if we have binary node states, hop distance of any node regarding state ‘1’ is the smallest number of hops needed from that node before we can find a node with state ‘1’. If the considered node itself has state ‘1’, then its hop distance is zero. Therefore, in a scenario where contagion spreads, the immediate neighbours of the percolated nodes will have smaller hop distances with respect to the percolation state. We will use hop distance 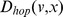 as a simple contrasting measure to percolation centrality in our simulations.

### Percolation centrality based on random walks

As mentioned above, it is not always likely that contagion will spread along shortest paths in networks. Indeed, pathological infection is more likely to spread randomly, where a person who is a ‘contact’ to an infected person is vulnerable to infection with a certain probability. Therefore, following the definition of betweenness centrality based on random walks [26], percolation centrality can also be defined in terms of random walks:
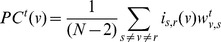
(16)where 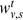 are the normalised weights (equation 6), and 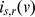 is the ‘current’ that flows through node 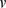 when the unit current is pumped between nodes 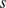 and 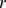 and all links are considered to have unit resistance. Note that the ‘current’ through node 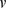 is to be calculated only to determine the fraction of shortest paths between nodes 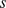 and 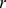 that, on average, pass through node 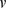, and as such this calculation will not affect the way the weight matrix is calculated (Alternatively, we can interpret the weights as the conductance of each link, and define percolation centrality then as simply the proportion of current between nodes 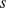 and 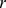 that passes through 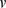.). Similarly, percolation centrality based on source and target weights, following equation 14, may be defined as
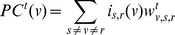
(17)where 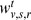 are the normalised weights (equation 15). We shall leave a detailed study of random walk based definitions of percolation centrality to future work.

### Implementation and computational complexity

The ‘straightforward’ implementation of a betweenness centrality algorithm would run in 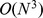 time [25], [26]. We implemented our percolation centrality measure as shown in equation 5 by modifying Brandes' fast algorithm for BC [25], which runs in 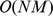 time. It can be shown that the extra calculations do not result in an order of magnitude increase in time complexity, and the algorithm still runs in worst case time 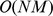. However, the definition of percolation centrality including target nodes (14) cannot be calculated in 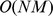 time. Brandes' algorithm achieves 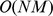 efficiency by iteratively counting the shortest paths from a given source node, and does not keep track of the targets. Therefore, calculating the percolation centrality with target nodes (equation 14) takes 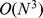 worst case time.

### An example with a simple model network

**Figure 1:**
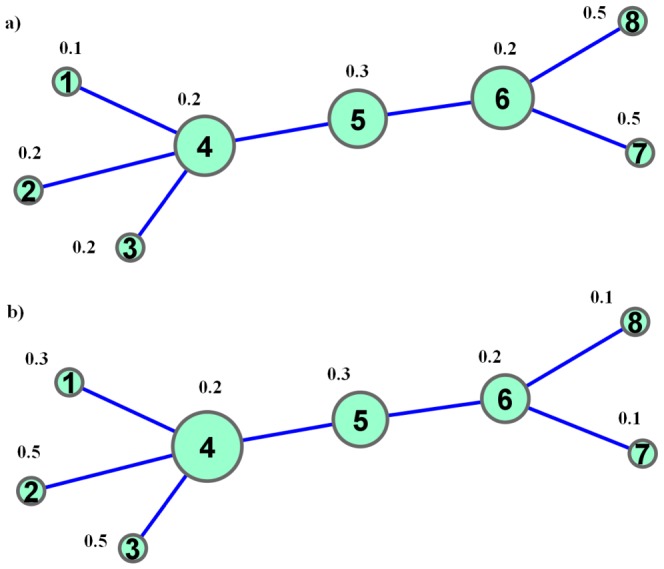
A simple network with 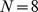. Note that in (a), the nodes in the right side of the network 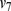 and 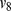 have high percolation states, whereas in (b), the nodes in the left side of the network 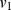, 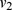 and 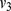 have high percolation states. The sizes of the nodes correspond to their percolation centrality values.

Let us now consider a simple example to illustrate the calculation of percolation centrality. Let us assume this is a network where partial percolation states are possible. Consider the Fig. 1 (a) where a simple network of eight nodes is shown, with percolation states ranging from 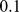 to 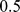 for each node. The same topology is repeated in Fig. 1 (b), with different percolation states. By inspection we could see that in Fig. 1 (a), the nodes at the right side, i.e the nodes 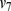 and 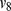, have the highest (partial) percolation state values, whereas in Fig. 1 (b), it is the nodes at the left side, i.e the nodes 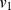, 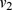 and 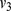 which have the highest percolation state values.

Now consider nodes 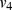 and 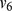. Both of these nodes are centrally located in terms of network ‘traffic’ and would have high betweenness centrality. If we calculated their percolation centrality based on Fig. 1 (a) (the calculation, which can be done manually but quite tedious, is left to the reader), we will see that node 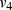 has the percolation centrality of 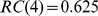 and 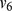 has the percolation centrality of 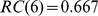. Even though their percolation centrality is also influenced by their topological placement, node 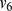 has slightly higher percolation centrality by virtue of being close to the nodes with higher values for percolation states (nodes 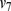 and 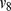). Whereas if we consider Fig. 1 (b), we will find that 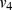 has the percolation centrality of 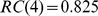 and 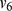 has the percolation centrality of 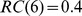. Here, while the topology remains the same, node 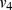 has much higher percolation centrality because it is now closer to the nodes with higher percolation state values (nodes 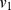, 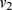 and 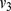).

This example demonstrates that percolation centrality of nodes in a fixed topology can vary significantly based on the percolated states of nodes in the network, and therefore the percolation centrality measure is quite dynamic unlike the centrality measures we have reviewed earlier.

## Results and Discussion

### Simulation of contagion spread using a simple real world network

**Figure 2:**
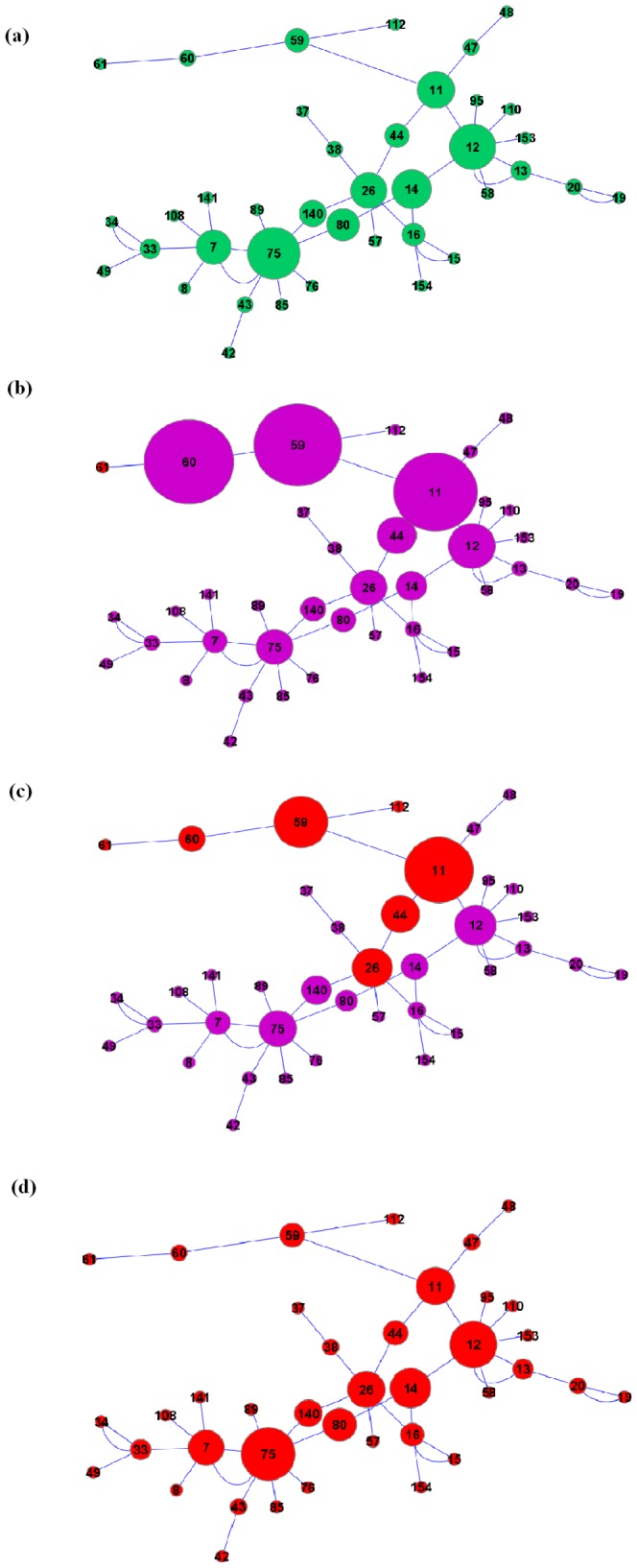
Betweenness and percolation centrality profiles of the Alberta model network with 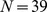, with node sizes matching the centrality values. (a) The betweenness centrality of nodes; independent of time. (b) The percolation centrality of nodes at 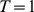. (c) The percolation centrality of nodes at 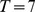. (d) Percolation centrality of nodes at 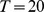. The infected nodes are highlighted in red.

We will use scale-free networks with hundreds of nodes to validate and exemplify the concepts presented so far, since, as mentioned earlier, most real world networks are scale-free networks. However, let us first look at a smaller real world network (with 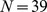) where tracking individual nodes as the contagion spread is possible and illustrative (The topology of the network we utilize is taken from the largest component of the Gonorrhoea outbreak study in Alberta, Canada [43], however we will use it here as a generic sample network, since our focus is not on developing centrality measures for epidemiology as such. It is rather on developing a generic centrality measure for contagion spread.). The network is shown in Fig. 2. We analysed percolation centrality of nodes in this network, by simulating the contagion spread for 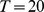 timesteps.

Since our aim is to demonstrate the utility of percolation centrality as a resource allocation tool, we will use a generic and simple contagion spread model. More specific spread models exist and can be used depending on the context of the application (For e.g., see [44] for an infection spread model in epidemiological scenarios). In our simulations, the contagion begins with a particular peripheral node.

**Figure 3:**
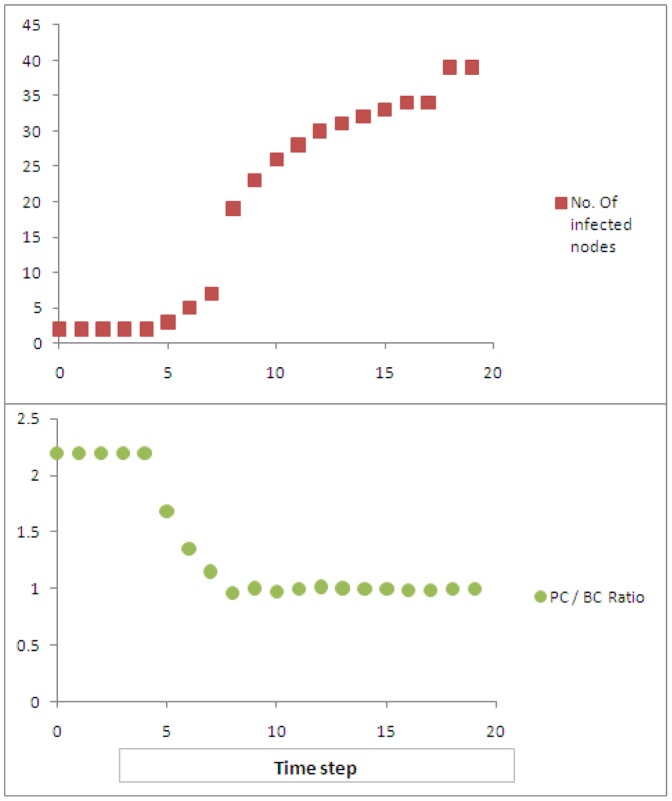
The number of percolated nodes, as well as the ratio of average PC and BC values, over time, for the Alberta model network. As the percolation becomes universal, this ratio settles around unity, as PC converges to BC for each node.

For example, Let us consider a specific simulation, in which a peripheral node 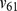 (the node at the top left hand side of the network in Fig. 2) is first to be fully percolated, 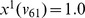, and all other nodes are not percolated, 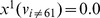, at time step 1. At each time step, the nodes are further percolated with a transmission probability 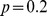 and no node becomes percolated without a direct contact with a percolated node (contagion spreads through links only). As the simulation progresses, the states of the percolated nodes are updated to 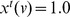. The number of nodes infected at each time step is shown in Fig. 3. We also trace how PC differs from BC at each time step, by considering the ratio of the averages between the measures for every node with non-zero betweenness. The ratio of averages over all 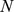 such nodes, 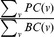, is also shown in Fig. 3 (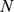 divides both the numerator and denominator and thus cancels out.).

We could see from Fig. 3 that at the beginning, 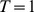, the PC of nodes, on average, is significantly different from BC. As the contagion progresses, around time 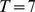, there is a sharp increase in the number of nodes fully percolated (establishing that this point corresponds to the well-known percolation phase transition is beyond the scope here). After this increase, the PC of nodes, on average, converges to the BC values. At the end of simulation, 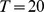, the PC of nodes, on average, are very similar to their BC counterparts.

This is further illustrated in Fig. 2, where we show the network with the node sizes corresponding to their centrality values (In the figure, the nodes with zero percolation/betweenness centrality are assigned a minimal size.). We observe that at 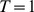, nodes 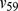 and 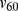 have the highest PC. Indeed, these nodes lie directly at the path of the potential spread of contagion, and therefore are critical for the contagion spread. The next highest node is node 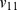, which has high overall BC, and is close enough to the percolated node. Node 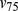 is not so large, since, while being centrally located, it is far from the percolated area.

At 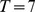, the contagion is about to ‘break through’ and the number of fully percolated nodes is going to rapidly increase. There are seven fully percolated nodes, highlighted in red. Here we could see that node 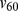 has lost its importance, and node 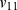 has the highest PC, due to its topological prominence as well as the percolation state of its neighbours. The PC values of the nodes are still significantly different from the respective BC values. Finally, at 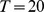, the contagion has spread to all nodes in the largest component, and nodes 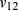 and 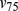 have the highest PC values. Indeed, the percolation centrality profile across the nodes at this stage is identical to the betweenness centrality profile of the network. Since all the nodes are fully percolated, the importance of nodes is measured based purely on topology, just like in betweenness centrality. Note that the size of nodes 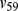 and 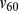 has reduced now, since they are on the periphery and no longer exclusively close to the contagion.

We may observe a number of interesting facts even in this arguably simple example. Firstly, percolation centrality is the most relevant when the spread of contagion is at its infancy, and remains very relevant until the number of percolated nodes goes through a sharp transition. Secondly, percolation centrality is not directly correlated to the distance of a node from its closest percolated neighbour: nodes which are centrally located (such as node 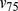 at time 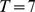) may have higher PC, even though they are further from the contagion than some other nodes. Crucially, at the early and critical stages of the contagion spread, the percolation centrality is not directly correlated to betweenness centrality either. Thirdly, when the network is mostly percolated, the percolation centrality profile across the nodes starts to closely resemble the network's betweenness centrality profile. Therefore, it is clear that the percolation centrality measure conveys information that is most relevant to a targeted early intervention. Consequently, in order to prevent the spread of contagion, one should target precisely the non-percolated nodes that have the highest percolation centrality. In the next section, we will study percolation centrality as a resource allocation tool while using larger scale-free networks as examples.

### Simulation experiments using scale-free and random networks

Scale-free networks are those networks that display similar topological features irrespective of scale. Such networks are described by power law degree distributions, formally specified as
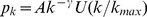
(18)where 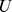 is a step function specifying a cut off at 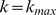. The degree distribution of scale-free networks can be specified by a number of parameters, including maximum degree 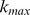, scale-free exponent 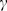, and the proportion of out-lier nodes 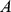. Most real world networks are scale-free networks, including technical, biological and social networks [1], [16], [45]–[49]. scale-free networks have been commonly used as model networks for infectious dynamics modelling [44], to represent road and air traffic networks [50] and to represent large scale computer networks, including Internet [1], [14], [51], [52]. Therefore, scale-free networks can be used as a justifiable model to simulate contagion spread scenarios.

We used a number of scale-free networks for our studies, where the network size was up to 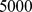 nodes. Let us consider a typical network with the number of nodes 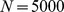, the number of links 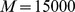, and 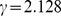. We will again use the generic spread model described above to simulate contagion spread. The contagion will start from a randomly selected node (either hub or peripheral), and this node is the first to be fully percolated, and all other nodes are not percolated, at time step 1. At each time step, the nodes are further percolated with a transmission probability 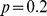 and no node becomes percolated without a direct contact with a percolated node (contagion spreads through links only). As the simulation progresses, the states of the percolated nodes are updated to 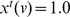.

**Figure 4:**
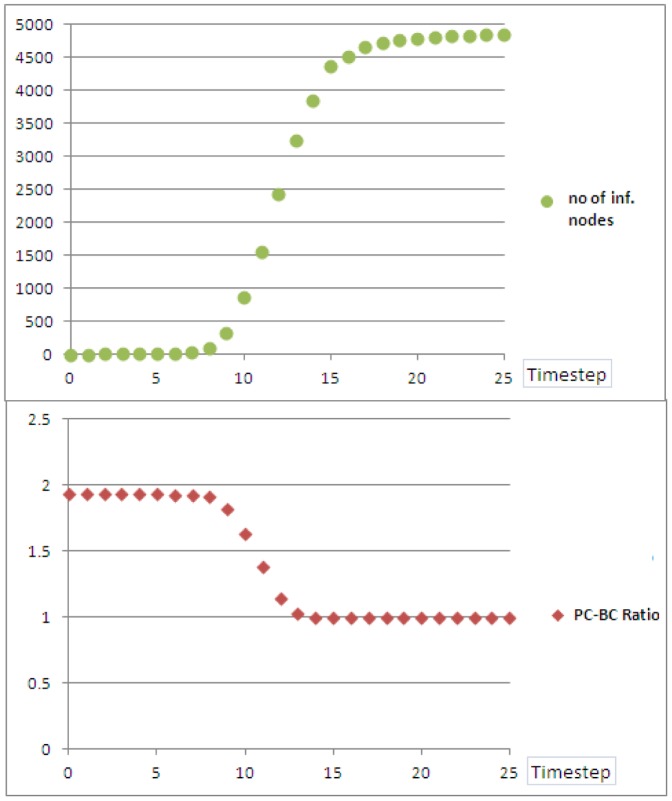
The number of fully percolated nodes, and the ratio of average PC and BC values over time, for a scale-free network with 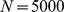. As the percolation becomes universal, this ratio settles around unity, as PC converges to BC for each node.

**Figure 5:**
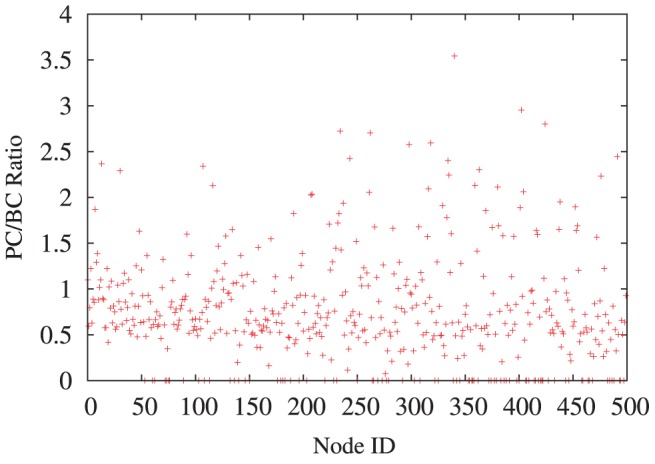
A typical run of the simulation, at timestep 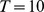, for a scale-free network with 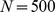. The Figure shows the 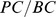 ratios against node ID. It could be noted that for some nodes, the PC is more than three times higher than the BC. The ratio is shown as zero if the betweenness of the node is zero.

The average number of nodes percolated vs timesteps is shown in Fig. 4 (averaged over 50 simulation runs). The ratio of averages, 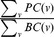, is also shown in the same figure. It could be seen that on average, the percolation ‘breaks through’ between timesteps 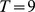 and 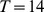, where there is a phase transition in the number of percolated nodes, as well as the ratio of averaged centrality measures. Once this phase transition is achieved, the ratio 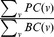 becomes close to unity. However, until the network is saturated with percolated nodes, the ratio 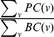 is higher than unity, meaning there is high diversity between percolation centrality and betweenness centrality. We confirmed this by analysing individual 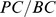 ratio profiles. For example, Fig. 5 shows these ratios (A network of size 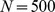 was used in this instance for clarity of figure.) against node IDs for a typical simulation run at time 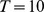 where we can see that, for some individual nodes, the 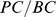 ratio can be as high as 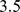, indicating significant variation between percolation and betweenness centralities at the critical part of contagion spread.

To demonstrate how percolation centrality could be utilized in real world as a resource allocation tool, we simulated ‘immunizing’ a certain percentage of nodes after a certain number of timesteps. The contagion could not spread through a node after it had been immunized. The nodes to be immunized were chosen based on one of the three following quantities:

1. Percolation centrality
2. Betweenness centrality
3. Hop distance

We immunized nodes after a certain percentage of nodes (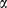) became fully percolated, and a certain percentage of nodes (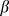) were ‘immunized’ at this timestep. The nodes to be immunized were selected by calculating a centrality measure (one of the three measures above) for all nodes, and ranking nodes top to bottom based on their values. The top 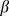 percentage of nodes were then ‘immunized’ (their node-states made permanently zero). Then we measured the number of timesteps it took, after intervention, for the network to be ‘saturated’ by percolation. Since due to topological effects it is possible that the network will never be completely ‘infected’, we took the network to be saturated by percolation if the number of fully percolated nodes passed a certain threshold. In the experiments described below, this saturation threshold is 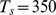 nodes (Even so, at some instances, the immunization may be so effective that the network may never reach this threshold. For practical purposes, therefore, we aborted such simulations thirty timesteps after immunization.).

Our simulation results are summarised in Appendix: Tables 1 and 2. The rows correspond to the percentage of nodes percolated before the intervention was made (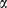), whereas the columns correspond to the percentage of nodes which were immunized (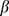). The number of timesteps it took, averaged over a number of simulations, for the network to be saturated by percolation is presented in the cells of each table. Each row has three sub-rows, corresponding to the centrality measure used for intervention: percolation centrality, betweenness centrality, and hop distance, in that order. For example, the entry of 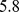 at the top left cell means that after two percent of the nodes were fully percolated, the top one percent nodes, selected by ranking nodes based on percolation centrality, were ‘immunized’, and despite this immunization, the percolation saturated the network in 5.8 timesteps on average. Similarly, the entry of 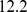 at the bottom right cell means that after twenty five percent of the nodes were fully percolated, the top twenty percent nodes, selected by ranking nodes based on hop distance, were ‘immunized’, and despite this immunization, the percolation saturated the network in 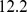 timesteps on average.

**Table 1:** A comparison between the average timesteps taken for saturation of percolation when the intervention is PC based, BC based or hop distance based. the network used was a scale-free network with 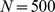.

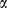 (rows) 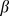 (columns)	1	2	3	4	5	6	7	8	9	10	11	12	15	20
2	5.8	6.0	6.2	7.8	10.2	10.4	11.8	15.4	15.2	17.8	22.2	24.6	27.8	30.0
	4.6	5.6	5.8	7.0	8.2	9.0	10.2	11.2	13.4	13.6	18.2	21.4	26.4	28.6
	6.4	6.8	7.2	10.2	14.4	18.8	22.4	25.2	28.6	28.4	30.0	30.0	30.0	30.0
3	4.6	5.8	7.0	8.8	9.6	10.6	11.0	13.4	14.2	18.8	22.6	24.2	28.4	29.2
	4.8	5.2	6.0	7.2	7.8	8.4	8.6	10.2	11.4	16.4	18.2	22.4	26.6	26.4
	6.2	6.4	9.2	10.8	13.0	15.6	17.6	18.8	24.4	27.6	29.2	30.0	30.0	30.0
4	4.2	6.0	6.6	7.8	9.0	10.8	11.2	13.4	15.0	16.8	19.6	24.0	27.2	29.2
	4.4	5.6	5.4	6.8	7.8	8.6	8.4	10.2	11.8	12.8	17.6	21.4	26.0	26.8
	4.0	5.6	6.2	8.0	8.4	11.0	10.4	14.2	14.4	18.8	22.4	27.8	30.0	30.0
5	4.8	5.0	5.8	7.0	8.6	9.4	10.0	11.2	13.2	14.8	18.6	20.2	28.6	28.8
	4.2	5.2	5.6	6.4	6.6	7.4	7.6	9.2	12.4	13.0	17.4	18.0	27.4	25.4
	3.8	4.2	5.4	7.2	7.8	10.2	10.4	11.0	12.2	15.2	15.8	22.6	25.4	29.6
6	3.8	4.8	6.2	7.4	8.6	9.2	10.2	11.8	14.4	16.0	19.2	25.6	27.4	28.2
	4.0	4.4	5.8	5.8	7.0	6.6	8.8	10.2	10.6	12.4	15.4	17.0	26.2	27.4
	3.6	4.2	6.0	6.6	7.8	9.4	10.6	11.2	14.8	15.2	17.2	20.2	28.2	28.4
7	4.0	5.8	6.4	5.2	8.8	9.4	10.2	12.6	15.0	14.8	17.6	22.2	26.8	27.4
	4.4	5.2	5.6	5.4	6.2	7.8	8.2	9.6	9.4	13.2	13.4	19.2	25.6	27.0
	3.4	3.6	5.4	5.2	7.6	8.8	10.0	13.2	14.2	15.0	16.6	18.6	23.2	27.8
8	3.6	4.4	5.4	6.4	7.8	9.2	10.6	11.8	12.0	16.6	18.4	23.8	28.0	28.2
	4.0	4.8	5.2	5.6	7.0	7.2	8.6	10.4	10.4	14.2	14.4	20.2	27.2	25.4
	3.2	4.0	5.2	6.0	7.2	8.6	9.4	10.4	11.6	13.2	14.4	18.2	20.2	28.4
9	3.4	4.8	5.2	7.8	10.4	9.4	11.6	13.4	14.0	15.6	18.8	20.6	27.4	27.2
	4.2	4.6	5.6	6.6	6.4	7.8	8.2	9.2	9.6	13.0	15.2	19.0	26.2	25.8
	2.4	2.8	5.0	4.4	4.2	8.4	9.2	8.8	13.0	11.0	13.4	16.8	21.8	27.4
10	3.2	3.8	4.2	5.6	7.2	7.8	7.8	9.0	10.2	13.2	16.6	16.4	26.2	26.4
	4.2	4.4	4.6	5.4	6.4	5.6	7.4	8.0	9.8	12.0	13.2	15.0	26.0	26.2
	2.2	2.8	3.2	4.0	4.8	5.0	4.8	7.2	8.4	10.2	11.0	12.2	13.2	18.4
11	3.0	4.0	5.0	6.4	8.8	8.4	10.2	11.8	12.0	15.6	14.8	18.2	26.4	26.6
	4.0	4.8	5.4	6.0	6.8	7.8	8.2	10.2	10.4	13.2	13.0	12.0	26.2	26.0
	1.8	2.0	1.8	3.4	5.6	6.4	5.8	9.8	8.4	9.8	11.8	10.4	13.4	15.2
12	2.8	3.0	4.0	4.4	5.6	5.4	7.8	8.0	9.8	10.2	11.4	13.4	25.3	26.2
	3.0	3.6	4.2	4.6	5.0	5.6	6.0	6.4	7.2	9.0	10.4	12.4	23.2	26.0
	1.6	2.2	1.6	1.4	4.2	2.8	2.8	4.2	3.6	8.2	9.6	10.6	12.0	15.6
15	2.6	2.8	3.0	4.2	3.8	5.2	6.0	6.8	7.8	8.8	11.2	12.0	18.2	20.0
	3.2	3.2	3.4	4.6	4.4	5.6	6.4	7.0	8.2	9.2	12.4	13.4	25.4	26.6
	1.4	1.6	1.6	2.8	3.0	4.2	3.8	5.8	6.0	8.0	9.2	8.8	14.2	13.8
20	2.2	2.0	3.2	3.8	4.2	4.8	5.6	5.8	6.6	7.6	9.0	11.2	14.4	17.2
	3.0	3.2	3.6	4.4	4.6	5.6	6.6	7.4	7.4	9.0	10.2	12.2	17.4	23.6
	1.2	1.6	1.4	1.4	2.0	3.8	3.2	3.8	4.0	6.2	5.8	8.0	10.6	14.4
25	1.6	1.8	2.2	2.8	3.2	2.8	4.4	6.6	6.6	7.2	7.8	9.6	13.4	16.8
	2.2	2.4	3.0	3.2	3.4	4.6	5.4	7.2	7.6	8.2	8.0	10.4	22.6	23.2
	1.4	1.2	1.2	1.2	1.6	2.0	2.6	3.8	3.4	3.6	3.2	4.4	5.8	12.2

Table 1 in this appendix summarises several thousand simulation experiments with scale-free networks and the obtained results. The rows correspond to the percentage of nodes percolated before the intervention was made (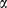), whereas the columns correspond to the percentage of nodes which were immunized (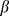). The number of timesteps it took, averaged over five simulations each, for the network to be saturated by percolation is presented in the cells of the table. Each row has three sub-rows, corresponding to the measures used for intervention: percolation centrality, betweenness centrality, and hop distance, in that order. All in all, the table presents the results of 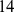
×
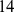
×
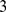
×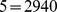 simulation experiments (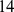 values of 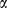 and 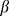, 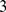 ways to measure centrality, repeated 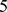 times).

**Table 2:** A comparison between the average timesteps taken for saturation of percolation when the intervention is PC based, BC based or hop distance based. the network used was a random network with 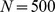.

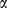 (rows) 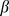 (columns)	1	2	3	4	5	6	7	8	9	10	11	12	15	20
2	4.42	4.64	4.12	4.34	4.42	4.64	4.56	4.32	4.78	4.12	5.12	6.24	5.82	7.24
	3.02	3.30	3.36	3.42	3.78	3.98	4.00	4.12	4.04	4.08	4.96	5.04	5.24	6.08
	5.64	5.84	5.92	5.96	6.04	6,32	6.86	6.78	6.96	7.02	6.98	7.34	9.22	10.36
3	4.32	4.64	4.84	5.26	4.48	5.24	4.48	5.62	6.24	7.18	6.28	6.84	7.22	7.04
	3.42	3.72	3.56	3.92	3.90	4.44	4.46	4.88	5.22	5.68	6.00	6.42	6.78	6.66
	4.28	4.92	4.82	5.50	4.36	5.38	5.58	5.92	6.36	8.42	8.00	6.76	8.48	10.22
4	4.16	4.22	4.82	5.04	4.84	4.26	4.44	4.58	5.02	5.04	5.18	5.26	5.54	5.98
	3.86	3.98	4.12	4.04	4.22	4.12	4.08	4.22	4.48	4.78	4.88	5.02	5.24	5.30
	4.10	4.04	4.36	4.80	4.82	4.22	4.08	4.34	4.56	5.28	5.16	6.34	5.22	7.88
5	4.04	4.22	4.18	4.32	4.44	4.56	4.66	4.68	4.62	4.76	4.80	4.82	4.98	4.86
	4.14	4.18	4.24	4.28	4.36	4.40	4.48	4.52	4.58	4.66	4.70	4.78	4.84	4.86
	3.88	4.14	4.04	4.22	4.34	4.26	4.58	4.60	4.60	4.72	4.94	4.78	4.80	4.84
6	3.8	3.92	4.10	4.08	4.24	4.72	4.46	4.52	4.78	4.80	4.82	4.86	4.90	4.94
	3.74	3.96	4.04	4.06	4.18	4.38	4.44	4.50	4.62	4.74	4.78	4.82	4.86	4.90
	3.76	3.84	3.98	3.96	4.06	4.14	4.44	4.32	4.52	4.60	4.76	4.72	4.86	5.88
7	3.68	3.74	3.80	3.84	4.20	4.14	4.32	4.36	4.56	4.60	4.72	4.68	4.78	4.80
	3.60	3.76	3.78	3.80	3.92	4.04	4.20	4.28	4.44	4.56	4.54	4.66	4.74	4.78
	3.24	3.32	3.38	3.40	3.62	3.58	3.88	3.78	3.96	4.04	4.08	4.22	4.18	4.34
8	3.24	3.64	3.68	3.70	3.72	3.88	3.92	3.98	4.22	4.42	4.34	4.48	4.56	4.60
	3.44	3.56	3.70	3.68	3.70	3.92	3.90	3.94	4.12	4.28	4.32	4.44	4.50	4.54
	3.04	2.98	3.06	3.12	3.24	3.46	3.48	3.58	3.66	3.70	3.84	3.92	4.02	4.06
9	3.26	3.28	3.34	3.46	3.42	3.56	3.88	3.78	4.18	4.28	4.34	4.46	4.50	4.52
	3.28	3.34	3.38	3.44	3.48	3.52	3.68	3.74	3.86	3.96	4.26	4.48	4.42	4.48
	2.68	2.74	2.86	2.74	3.02	3.00	3.06	3.18	3.08	3.24	3.38	3.40	3.64	3.88
10	3.2	3.18	3.24	3.46	3.50	3.66	3.68	3.72	3.88	4.06	4.18	4.22	4.36	4.40
	3.12	3.20	3.22	3.48	3.46	3.58	3.64	3.76	3.80	3.92	4.20	4.18	4.26	4.34
	2.44	2.46	2.58	2.64	2.72	2.80	2.66	2.96	3.12	3.16	3.42	3.34	3.58	3.48
11	2.92	3.10	3.12	3.28	3.42	3.46	3.58	3.66	3.70	3.82	3.92	3.96	4.02	4.22
	2.98	3.14	3.16	3.24	3.48	3.42	3.60	3.62	3.68	3.70	3.88	3.90	3.96	4.34
	2.22	2.32	2.34	2.48	2.50	2.68	2.64	2.62	2.78	2.80	2.92	2.94	3.06	3.16
12	2.60	2.66	2.78	3.02	3.08	3.12	3.24	3.44	3.48	3.56	3.60	3.64	3.88	3.86
	2.56	2.70	2.82	2.94	3.12	3.08	3.16	3.52	3.46	3.54	3.58	3.60	3.72	3.84
	2.02	2.04	2.18	2.26	2.38	2.34	2.44	2.54	2.62	2.78	2.86	2.90	2.94	2.96
15	2.26	2.40	2.52	2.68	2.76	2.84	2.90	2.98	3.04	3.08	3.14	3.16	3.24	3.32
	2.36	2.44	2.56	2.74	2.74	2.78	2.92	2.96	3.08	3.06	3.24	3.10	3.18	3.30
	1.86	1.80	1.88	1.98	2.04	2.22	2.12	2.34	2.94	2.46	2.58	2.60	2.12	2.64
20	2.02	2.14	2.08	2.16	2.18	2.34	2.26	2.66	2.64	2.76	2.80	2.90	2.96	2.94
	2.06	2.18	2.20	2.24	2.28	2.22	2.36	2.70	2.66	2.80	2.78	2.92	2.94	2.90
	1.46	1.48	1.50	1.68	1.54	1.76	1.84	1.92	1.96	2.08	2.10	2.34	2.24	2.40
25	1.80	1.84	1.86	1.90	1.92	1.90	1.94	1.92	2.02	1.98	2.12	2.24	2.30	2.38
	1.84	1.88	1.90	1.94	1.96	1.98	1.92	2.00	2.06	2.08	2.18	2.30	2.44	2.48
	1.40	1.34	1.64	1.58	1.70	1.86	1.84	1.90	1.96	1.98	2.04	2.00	2.12	2.14

Table 2 summarises several thousand simulation experiments with random networks and the obtained results. In this case, given random nature of the experimental networks, we repeated the experiment for each combination of 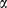, 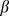, and a centrality measure 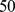 times. The rows denote the percentage of nodes percolated before intervention was made (parameter 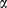), whereas the columns denote the percentage of nodes which were ‘immunized’ against the spread (parameter 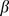). The first, second, and third sub-rows denote timesteps related to PC based, BC based and hop distance based intervention respectively.

**Figure 6:**
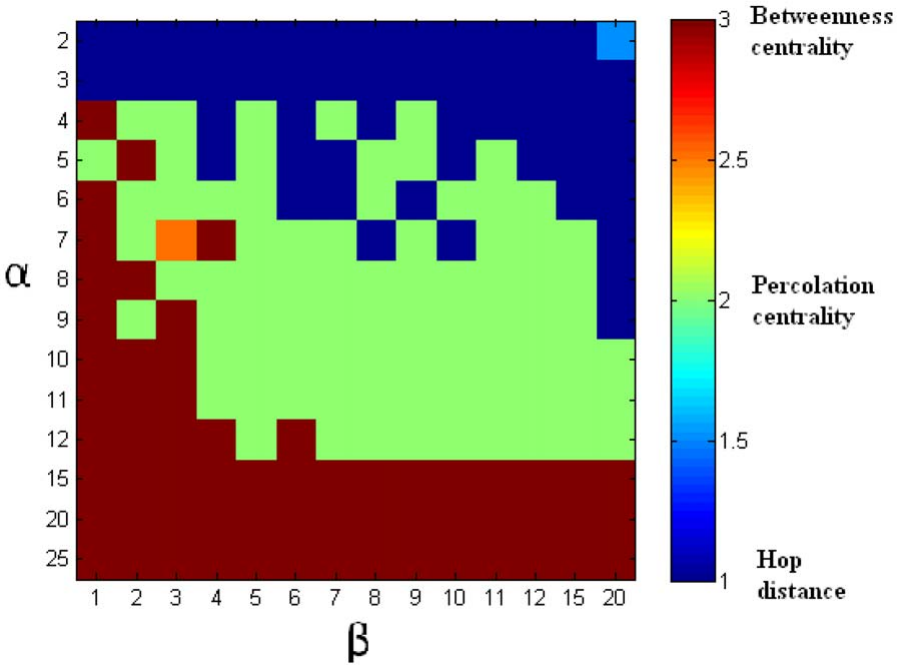
Scale-free network: the ranges of 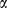 and 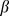 for which the various centrality measures show the best performance. This figure corresponds to Table 1. Red: best performance by betweenness centrality based intervention (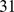
%). Green: best performance by percolation centrality based intervention (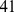
%). Blue: best performance by hop distance based intervention (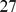
%). Intermediate colours represent ties (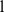
%).

**Figure 7:**
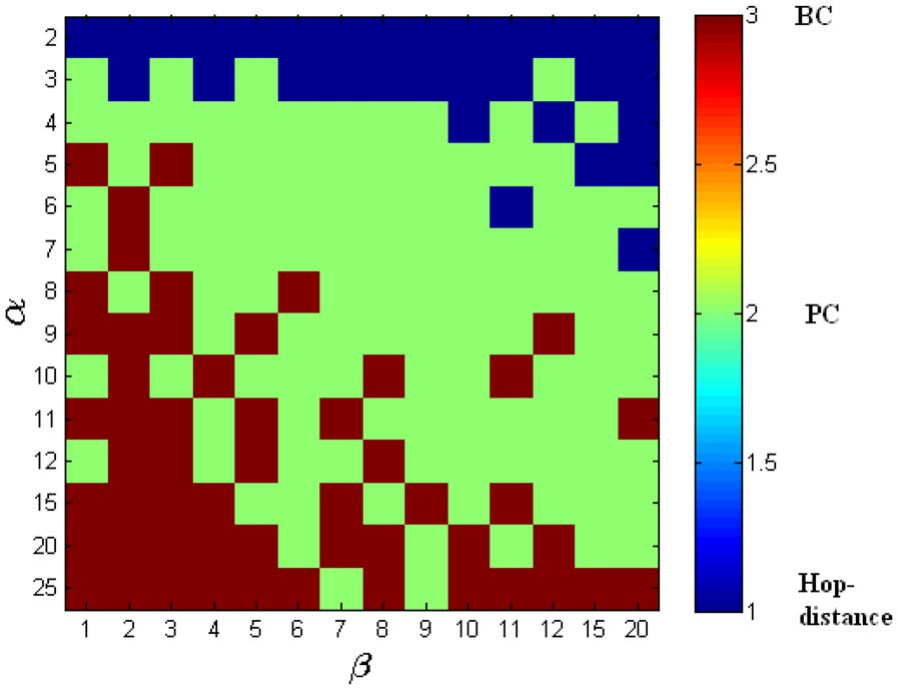
Random network: the ranges of 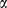 and 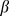 for which the various centrality measures show the best performance. This figure corresponds to Table 2. Red: best performance by betweenness centrality based intervention (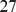
%). Green: best performance by percolation centrality based intervention (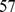
%). Blue: best performance by hop distance based intervention (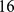
%).

We investigated two topologies: scale-free networks and Erdös-Renyi random networks. Erdös-Renyi random networks were chosen for our experiments to contrast them with scale-free networks, and to reflect common real-world topologies which are not scale-free, e.g. non-scale-free networks of towns and motorways [53], [54]. The results for scale-free networks are presented in Table 1 and Figure 6, while Table 2 and Figure 7 summarise experiments with Erdös-Renyi random networks.

Figure 6 shows the matrix of 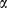 and 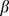 values used in our simulation. For a given 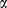 and 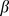, the colour of the matrix corresponds to the measure which returned the best performance. For example, if 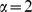 and 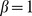, we could see from Table 1 that percolation centrality based intervention resulted in an average 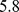 timesteps before the network was saturated. Similarly, betweenness centrality based intervention resulted in an average 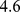 timesteps, and hop distance based intervention resulted in an average 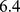 timesteps. Therefore, hop distance was the intervention method which delayed the saturation of percolation furthest, and the corresponding cell in the matrix is shown in blue to indicate this. A green cell shows that percolation centrality was the most useful measure, and a red cell shows that betweenness centrality was the most useful measure. Intermediate colours show there is a tie (same highest average time was obtained for more than one centrality measure).

It could be seen from Table 1 (Appendix) and Fig. 6 that, there are certain circumstances in which percolation centrality becomes a useful resource allocation tool, resulting in longer times for the network to get completely percolated. Particularly, if 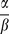 ratio is high (this means either 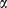 is high or 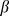 is low, and it is easier for the contagion to spread further), then betweenness centrality is the most effective tool for resource allocation. This makes intuitive sense because if we are too late to intervene (high 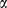) or have too few resources (low 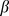), protecting the ‘core’ of the network from contagion will be our best strategy. On the other hand, if 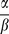 ratio is low (this means either 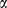 is low or 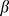 is high, and it is hard for the contagion to spread further), then hop distance becomes the most effective tool for resource allocation. Again this is not surprising, since if we have sufficient resources (high 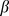) and sufficient time (low 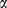), we can ring-vaccinate all the nodes around percolated nodes.

It is when the ratio 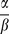 is in the medium ranges, that percolation centrality becomes the best resource allocation tool. Indeed, it can be seen that in the ‘critical’ middle realms of both 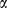 and 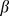, in which the percolation is often realistically detected, percolation centrality based intervention has the longest saturation times, and thus the highest chance of slowing down the percolation process. This is the case for 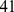
% of the cells in Fig. 6, for the range of 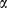 and 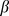 that we have selected. While this percentage is subject to the selection of 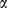 and 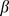, the general pattern is clear. As such, the measure of percolation centrality of nodes can be effectively used as a resource allocation tool.

It can be easily seen that our conclusions hold for ER random topology as well: Figure 7 and Table 2 show that for the middle range of ratio 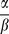 allocating the resources according to the percolation centrality is again the most effective way to delay percolation.

## Conclusions

We introduced a new centrality measure (percolation centrality) to analyze the importance of nodes during percolation in networks. We demonstrated that when a network is fully percolated (that is, all nodes have the same state), our measure reduces to betweenness centrality. However, when only some nodes are (partially or fully) percolated, betweenness and other existing centrality measures can be ineffective in identifying the relative impact of nodes on further percolation (e.g., on the spread of infection). On the contrary, percolation centrality becomes a useful measure precisely in these scenarios when an early intervention is warranted. Percolation centrality in its basic form can be calculated in 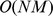 time, thus including no significant increase in time complexity from standard betweenness centrality. We stress that percolation centrality is introduced here as a generic measure applicable in the context of *any* contagion spread, ranging from computer virus proliferation to information spread in social networks. We also note that, unlike weighted betweenness measures, percolation centrality is dynamic and has relatively low computational complexity. We have also analytically derived some simple relationships between percolation centrality and betweenness centrality for extreme cases. Firstly, percolation centrality averaged over all possible single contagion sources was shown to reduce to betweenness centrality. Secondly, it was shown that if all nodes are infected (or partially percolated to the same extent), then percolation centrality also reduces to betweenness centrality.

We used a simple network of 39 nodes obtained from a contact network study to demonstrate how percolation centrality could be utilised. We simulated a contagion spread, and used percolation centrality to identify critical nodes. Since most contact networks could be modelled as scale-free networks, we also utilized scale-free networks to demonstrate the measurement of percolation centrality, and compared this with results from ER random topologies.

We demonstrated that percolation centrality can be used as a resource allocation tool. Particularly, using simulated scale-free and random networks, we showed that allocating resources according to the percolation-centrality based ranking is most effective when the stage of percolation and the amount of resources available are at a medium level. It was shown that when the percolation process is at its infancy, hop-distance based measures, such as ring vaccination, are most effective to contain the percolation. If the percolation process has affected the node states of a majority of nodes, then betweenness centrality based ranking is most effective. Similarly, if we have extremely limited resources to ‘immunize’ nodes to percolation, betweenness centrality based choices are most effective, and on the other hand, if we have considerable resources, hop distance based choices are most effective. Percolation centrality based ranking becomes an effective tool at the critical stage where the percolation is just about to break through, and we have a considerable but limited amount of resources to immunize nodes against the spread.

Percolation centrality lends itself to the many extensions to which betweenness centrality has been subjected in the past. For example, a definition of percolation centrality based on random walks can be proposed, where the ‘walking agents’ could be given weights based on source node states. Furthermore, percolation centrality could be analyzed for both source and target node states. As such, the concept has great potential for further research.
